# Structure-aware protein solubility prediction from sequence through graph convolutional network and predicted contact map

**DOI:** 10.1186/s13321-021-00488-1

**Published:** 2021-02-08

**Authors:** Jianwen Chen, Shuangjia Zheng, Huiying Zhao, Yuedong Yang

**Affiliations:** 1 School of Data and Computer Science, Sun Yat-Sen University, Guangzhou, China; 2 Sun Yat-Sen Memorial Hospital, Sun Yat-Sen University, Guangzhou, China; 3 Key Laboratory of Machine Intelligence and Advanced Computing (Sun Yat-Sen University), Guangzhou, 510000 China

**Keywords:** Protein solubility prediction, Graph neural network, Predicted contact map, Deep learning

## Abstract

Protein solubility is significant in producing new soluble proteins that can reduce the cost of biocatalysts or therapeutic agents. Therefore, a computational model is highly desired to accurately predict protein solubility from the amino acid sequence. Many methods have been developed, but they are mostly based on the one-dimensional embedding of amino acids that is limited to catch spatially structural information. In this study, we have developed a new structure-aware method *GraphSol* to predict protein solubility by attentive graph convolutional network (GCN), where the protein topology attribute graph was constructed through predicted contact maps only from the sequence. *GraphSol* was shown to substantially outperform other sequence-based methods. The model was proven to be stable by consistent ${\text{R}}^{2}$ of 0.48 in both the cross-validation and independent test of the *eSOL* dataset. To our best knowledge, this is the first study to utilize the GCN for sequence-based protein solubility predictions. More importantly, this architecture could be easily extended to other protein prediction tasks requiring a raw protein sequence.
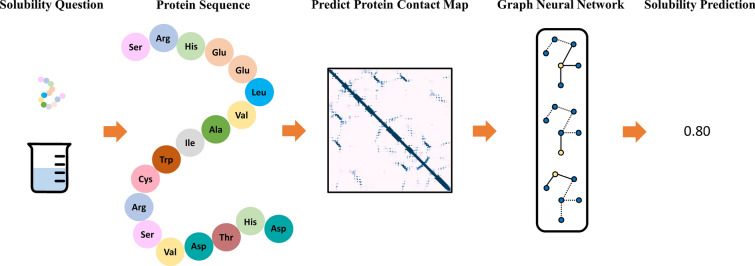

## Introduction

Over the past 20 years, recombinant protein had played a vital role in biotechnology and medicine, including novel therapeutic protein drugs and antibodies [1]. Recombinant proteins are mostly produced by genetic engineering in Escherichia coli (*E.coli*) [2]. However, low solubility and activity of proteins expressed by *E.coli* limited the production efficiency even though the standard workflow and logical strategies have been widely deployed in biopharmaceutical industries [1]. According to statistics, over 30% of recombinant proteins are not soluble [3], 33–35% of all expressed non-membrane proteins are insoluble, and 25–57% of soluble proteins are prone to aggregate at higher concentrations [4]. Moreover, the heterologous expression often suffers from low levels of production and insoluble recombinant proteins forming inclusion bodies. Therefore, the protein solubility plays an important role in the production of proteins for the biotechnological and pharmaceutical industries.

To enhance the performance of recombinant proteins, many experimental technologies have been developed, e.g. directed evolution, immobilization, designing better promoters, optimizing codon usage, and changing culture conditions including media and temperature [5, 6]. However, such empirical optimizations are labor-intensive and time-consuming. A precise computational model is highly desired so that protein solubility can be effectively predicted. Theoretically, given an exact experimental condition (i.e. temperature, expression host, etc.), the solubility is determined mainly by its primary structure that is decided by the sequence [3]. To this end, two types of computational approaches have been proposed to predict the protein solubility: physical-based and machine/deep learning-based methods.

In terms of the physical-based techniques, most works [7, 8] focused on making use of extensive molecular dynamics simulations to evaluate the free energy difference between aggregation and solution phases. However, these methods are usually of limited accuracy due to difficulties in evaluating the conformational entropy and solvent contributions. Furthermore, these atom-level methods are sensitive to structural fluctuations and can’t process protein flexibility well [9].

For the machine/deep learning techniques, several sequence-based methods have been developed for protein solubility prediction including *PROSO II* [10], *CCSOL* [5], *SOLpro* [11], and the scoring card method (*SCM*) [12]. The majority of these methods adopted the support vector machine (*SVM*) [13] as the core discriminative model on biologically relevant handcrafted features from protein sequences to discriminate the soluble and insoluble proteins. The newly proposed method, *PaRSnIP* [14] was developed by identifying correlations of protein solubilities positively with fractions of exposed residues while negatively with tri-peptide stretches containing multiple histidines. *Protein-Sol* [15] employed a different combination of feature weights in averaging the sequence-based local and global properties.

With the development of deep learning techniques, many end-to-end methods have been developed. *DeepSol* built a convolutional neural network (*CNN*) [16] to construct non-linear high-dimensional vector spaces with essential information for predicting protein solubility [17]. *ProGAN* generated extra data from a Generative Adversarial Networks (*GAN*) [18] that had been learned by the training set to improve the final performance [19]. *TAPE* [20] and *SeqVec* [21] trained a general model from a large protein database and provided a pretrained embedding for other protein downstream tasks. However, these methods are mostly based on Long Short-term memory (*LSTM*) [22] or Transformer [23] and didn’t utilize spatial information of protein molecules. Though our recent studies indicated that the protein structure could be well represented and the contacted structural information could be implicitly included by the residue-pairwise distance matrix through CNN [24, 25], the information aggregated from regular Euclidean space could not fully interpret the relations between residues in contact.

In the past few years, the Graph Neural Network (*GNN*) was raised to represent the protein structure in various deep learning-based methods and had made successes in properties prediction [26–28]. However, these methods demand experimentally obtained 3D structures that are hard to acquire for aggregation proteins and thus are not appropriated for sequence-based protein solubility prediction.

With recent developments in protein structure prediction, the prediction of protein contact map has been greatly improved according to the critical assessment of protein structure prediction (CASP) [29], which brought another way to get accurate contact structural information without using the protein 3D structures. There are quite a few predictors solved this protein by evaluating the residue-residue contact [29–31], for example, Hanson et al. [32] had developed a novel sequence-based method in predicting protein contact map and reached the state-of-the-art performance, which aimed to capture these deep, underlying relationships between residue-residue pairs in spatial dimensions for protein ‘image’ at each layer. Compared to other algorithms, the predicted protein contact map integrates all their advantages so that it can represent 2D structural features directly in high accuracy, enabling the construction of accurate protein graphical representations from protein sequence. And a similar constructed structure also helps us evaluate the predicted performance when compared to the possible experimental structure.

Inspired by these new development tools, we proposed a novel structure-aware method *GraphSol* for protein solubility prediction from the sequence by combining predicted contact maps and graph neural networks. The predicted contact maps were employed to construct protein graphs, and the attentive-based graph convolutional network made the predictions through mapping the nodes (amino acids) embedding to the graph full content embedding. We performed our model in the *eSOL* database [33] and obtained state-of-the-art performance. To the best of our knowledge, this is the first study to make sequence-based solubility prediction for proteins through graph neural networks. Moreover, such architecture could be easily applied to extensive tasks on proteins, e.g. protein function prediction, protein–protein interaction prediction, protein folding, and drug design.

## Methods

### Overview

In this study, we convert the protein solubility prediction task as a graph-based regression problem. Given a protein sequence that consists of $L$ amino acids, the whole protein could thus be expressed as a topological attributed graph $G(F, E)$, with $F$ for the feature set of all residues (nodes) and $E$ for the residual contacts (edges) according to predicted protein contact map. Our task aims to learn a mapping function $f\left(\cdot \right)$ that inputs with predicted residual features and contact map and outputs predicted solubility with continuous scores between $\left[0,1\right]\in {\mathbb{R}}$ i.e. $f:G\left(F,E\right)\to [0,1]$. In this work, $f\left(\cdot \right)$ is a graph convolutional network model that aggerates nodes and edges information on the irregular graph.

### Datasets

#### *eSOL* dataset

To train our model, we employed the *eSOL* dataset from the previous study [34]. For completeness, we briefly describe the procedure to produce the dataset. The whole solubility database of ensemble *E.coli* proteins was downloaded from the *eSOL* website [33], where the solubility was defined as the ratio of the supernatant fraction to the total fraction in the physiochemical experiments named PURE [35]. The 4132 proteins were firstly mapped to the *NCBI* database by gene names, and 3,144 samples were returned. We further pruned out all sequences using a strict standard that had a sequence identity $\ge 25\%$ or E value $\le 1\text{e}-6$ according to previous observation [36], and the final data included a total of 2737 protein sequences. From the dataset, 75% (2052 proteins) were randomly selected as the training set, and the remaining 25% (685 proteins) were used as the independent test.

#### *S. cerevisiae* dataset

For an external independent test, we selected another protein dataset collected by [9] from the *S. cerevisiae*. This dataset was derived by including 108 proteins having corresponding 3D structures. The solubilities were also measured by the cell-free expression called *PURE* [35] in the same external condition to reduce the influence caused by the environment.

### Protein representation

#### Node features

We devised four groups of protein features that were used to train the GraphSol predictor model.

##### Blosum62

Instead of one-hot encoding, we have encoded residues by Blosum62 [37], which is a widely used 20 × 20 matrix for substitutions between 20 standard amino acid types according to alignments of homologous protein sequences. The blosum62 was shown to outperform simple one-hot encoding (results not shown), as also indicated in our previous study [38].

##### Physicochemical properties

We utilized a set of 7 physicochemical properties for amino acid types (*AAPHY7*) [39]. These features include steric parameters, hydrophobicity, volume, polarizability, isoelectric point, helix probability, and sheet probability.

##### Evolutionary information

Evolutionarily conserved residues may contain the motifs related to protein properties (such as solubility) in biological sequences [40]. Here, we employed the position-specific scoring matrix (*PSSM*) and the Hidden Markov matrix (*HMM*). To be specific, the *PSSM* profile was produced by *PSI-BLAST* v2.7.1 [41] with the *UniRef90* sequence database after 3 iterations. The *HMM* profile was produced by *HHblits* v3.0.3 in aligning the *UniClust30* profile *HMM* database [42] with default parameters.

##### Predicted structural properties

Predicted structural properties are highly related to solubility in the previous study [17]. Herein, we derived the predicted structural features from *SPIDER3* [43], one of the most accurate predictors. The feature group includes 14 features: (1) three probability values respectively for three secondary structure states (*SS3*), (2) Relative Solvent-Accessible Surface Area (*ASA*), (3) eight values for the sine/cosine values of backbone torsion angles (*phi, psi, theta, tau*), and (4) Half-Sphere Exposures based on the ${\text{C}}_{{{\upalpha }}}$ atom (*HSE-up* and *HSE-down*).

Finally, these feature groups constructed the node feature matrix $\text{X}\in {\mathbb{R}}^{L\times 94}$ with $L$ representing the length of a protein sequence. Table 1 listed all node feature groups with their dimensions. All data were standardized to zero mean and unit variance before input into the neural network.

**Table 1:** Node features and dimensions

Group	Node features	Names	Dimensions
1	Blocks substitution matrix	BLOSUM62	20
2	Physicochemical properties	AAPHY7	7
3	Position-specific scoring matrix	PSSM	20
	Hidden markov matrix	HMM	30
4	Structural properties predicted by *SPIDER3*	SPIDER3	14

#### Edge features

In order to construct the edges for the protein attribute graph representation, we make predictions of the protein contact map from a sequence by *SPOT-Contact* [32], which outputs the possibilities to form contacts between all residue pairs in one protein. In default, the graph is a fully connected graph constructed with each edge valued as the predicted contact probability of the corresponding residue pair. As the actual number of contacts in a protein is approximately proportional to the protein sequence length, we also test constructing the protein attribute graph by setting up edges for ${\upalpha }\times \text{L}$ residual pairs with the highest predicted contact probability, as also used in the CASP [29]. The ${\upalpha }=1\sim 7$ was utilized as suggested by [44]. Herein, we tested two schemes to construct each selected edge by setting the value as “1” (discrete) and the predicted contact probability (continuous), respectively. The 2-hop neighbored residues in a sequence are always connected with the value “1”. Notably, though the fully connected graph (default mode) was shown to perform the best according to our results, the partial edges decrease the computational and memory complexity from $\text{O}({\text{L}}^{2})$ to $\text{O}(\text{L})$.

### Deep learning framework

**Fig. 1:**
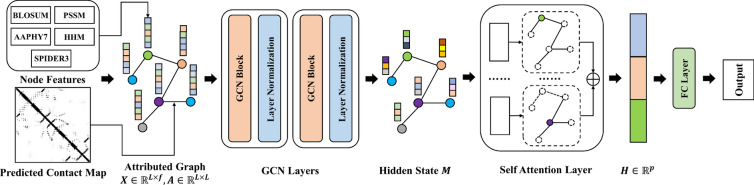
The overall framework of the GraphSol model. The compiled node features (X) and contact maps (A) were utilized to construct the GCN networks, which were convoluted by two GCN blocks and layer normalizations for hidden states (M). The hidden states were converted by the self-attention layer as a fixed length of the vector (H), which were input to the MLP to make the final prediction

Our graph-based model consists of three parts. As shown in Fig. 1, the first part is a graph convolution network (GCN), which aggregates protein structural information from its nodes and edges during iterations. The second part is a self-attention pooling layer, which transforms the node hidden state with varied sizes to the graph representation vector with a fixed size. Finally, this fix-sized vector goes through some full connection layers to predict the protein solubility.

### Graph convolution network

Given a protein sequence with $L$ amino acids, the protein is represented by the feature matrix $X\in {\mathbb{R}}^{L\times f}$ for nodes and contact matrix $A\in {\mathbb{R}}^{L\times L}$ for edges with $f$ as the dimension of features for nodes. Our graph convolution network takes the following formula [45]:

1 $$\begin{array}{c}{G}^{\left(l+1\right)}=\sigma \left({\stackrel{\sim }{D}}^{-1}\stackrel{\sim }{A}{G}^{(l)}{W}^{(l)}\right),\end{array}$$

where $\stackrel{\sim }{A}=A+{I}_{L}$ is the adjacency matrix by adding the edge matrix *A* determined by the predicted contact map and the identity matrix ${I}_{L}$ for self-loops. $\stackrel{\sim }{D}\in {\mathbb{R}}^{L\times L}$ is a diagonal degree matrix with ${\stackrel{\sim }{D}}_{ii}={{\sum }_{k}\stackrel{\sim }{A}}_{ik}$ that is used to normalize $\stackrel{\sim }{A}$ to sum up to 1.0 in each row. ${G}^{(l)}\in {\mathbb{R}}^{L\times f}$ is the activation hidden matrix in the ${l}^{th}$ layers with the initial state ${G}^{(0)}=X$ here. ${W}^{(l)}\in {\mathbb{R}}^{f\times {f}^{^{\prime}}}$ is a weight matrix of layer-specific trainable parameters to map the iteration to a lower dimension rich-information space with a size of ${f}^{^{\prime}}$. $\sigma$ denotes a nonlinear activation function and we use the $\text{ReLU}\left(\cdot \right)$ function here. After each GCN layer, a normalization layer is added to accelerate the convergence of the GCN layers as well as reduce the overfitting problem. The final output of these GCN layers are integrated as

2 $$\begin{array}{c}{M}=\left({v}_{1},{v}_{2},\dots ,{v}_{L}\right),\end{array}$$

where ${v}_{i}$ is a $p$ dimensional vector token embedding for the ${i}{th}$ node. As a result, $M$ is a 2D matrix to integrate all token embeddings with ${\mathbb{R}}^{L\times p}$.

### Self-attention pooling

Note that the output matrix $\text{M}$ is dependent on the protein length, which is a variable scale. To obtain a fixed size of protein representation, a readout transformation is essential to eliminate the size variance and sequence permutation variance [46]. Herein, we employ the self-attention mechanism [47], which computes the weight coefficients $T{\in {\mathbb{R}}}^{r\times L}$ with $r$ for the number of attention groups by:

3 $$\begin{array}{c}T=SoftMax\left({{\varvec{W}}}_{2}tanh({{\varvec{W}}}_{1}{M}^{T})\right),\end{array}$$

where ${M}^{T}$ is the transposition of ${M\in {\mathbb{R}}^{L\times p}}$.${{\varvec{W}}}_{1}\in {\mathbb{R}}^{q\times p}$ and ${{\varvec{W}}}_{2}\in {\mathbb{R}}^{r\times q}$ are two learned attention matrices with the hyper-parameters *q* and *r*. The *SoftMax* function standardizes each row of the computed weights, to sum up to 1. Intuitively, the *r* groups of attention coefficients assess the associations of each residue with the solubility from different views. Thus, we extract the overall features by multiplying T and M, and average all $r$ groups of attention coefficients for the final graph representation $H\in {\mathbb{R}}^{1\times p}$ by

4 $$\begin{array}{c}\\ H=\frac{1}{r}\sum_{k=1}^{r}{\left(TM\right)}_{k}\end{array}$$

### Multilayer perceptron

The output of self-attention pooling was input to the multilayer perceptron (MLP) to predict the solubility *S* by

5 $$\begin{array}{c}S=Sigmoid\left({\mathbf{W}}_{3}{H}^{\text{T}}+\text{b}\right),\end{array}$$

where ${\mathbf{W}}_{3}\in {\mathbb{R}}^{1\times \text{p}}$ is the weight matrix and $\text{b}\in {\mathbb{R}}$ is the bias item. The sigmoid function maps the value to $(0,1)$ for solubility prediction.

### Training and evaluation

#### Hyper-parameters tuning

Our model for solubility prediction includes multiple hyperparameters. We tested crucial hyperparameters and the range of values as follows:

##### GCN layers

A higher number of GCN layers means the wider and deeper information aggregated from the edge and node features. However, excessive layers would cause a decrease in final predicted accuracy due to vanishing gradients. Therefore, it is crucial to keep a balance between the layers and the algorithm complexity. We tested the following settings $(1,2,3,4)$ and found 2 GCN layers to be the optimal value after tuning on the validation sets.

##### GCN middle dimensions

These hyper-parameters control the channel dimensions in all stacking GCN layers including the final GCN layer. They influence the matrices that are transferred into self-attention pooling to identify the key soluble fragment of the protein. Therefore, we should construct a suitable size for matrices in liberating the rich-information regions as well as the distinguishability of different proteins. As a result, the optimal parameters are 64 dimensions for the last layer, and 256 for others.

##### Attention heads

The attention heads provide weight coefficients to focus on key residues for the solubility prediction, and different heads enable the attention of multiple regions from different views. We tested the number of attention heads from 1 to 10 and found 4 attention heads provided the best performance and the least calculation in the validations.

Besides, the models were trained for different epochs using Adam optimizer [48]. Additional file 1: Table S2 showed the optimal hyper-parameters by the grid search.

#### Cross-validation and independent test

We performed the fivefold cross-validation on the training dataset. That is, proteins in the training dataset were separated into five parts (folds). In each round four folds were employed to train a model that was evaluated on the left one-fold. This process was repeated five times, and the performances of five predictions were averaged as the validation performance. To reduce fluctuations by the random splitting of fivefold, we have split the training set with five random seeds and took an average of final performances. The validations were used to optimize all hyper-parameters. After fine-tuning the optimal hyper-parameters, a model was trained by all training dataset and independently tested on the two independent test datasets.

#### Evaluation indicators

The neural network was trained to minimize the root of mean squared error (RMSE), and the coefficient of determination (${\text{R}}^{2}$) was used to evaluate our models and optimize the hyper-parameters. Since many compared methods [5, 14, 15, 17] have been developed to classify whether a protein is soluble or not, we also separate all proteins into two classes by a threshold of 0.5 for the predicted and actual solubility. Statistically speaking, the definition of solubility mentioned before supports us regarded the soluble fraction of proteins as the soluble probabilities from other perspectives. Based on this setting, the models were evaluated by the area under the Receiver Operating Characteristic *(ROC)* curve (AUC), accuracy, precision, recall, and F1 defined as:

6 $$\begin{array}{c}Precision=TP/(TP+FP)\end{array}$$

7 $$\begin{array}{c}Recall=Tp/(TP+FN)\end{array}$$

8 $$\begin{array}{c}{F}_{1}=2\times (Precision\times Recall)/(Precision+Recall)\end{array}$$

where TP, FP, TN, and FN denote the numbers of true positives (soluble proteins), false positives (non-soluble protein predicted as soluble), true negatives, and false negatives, respectively.

## Results

### Performances on the fivefold cross-validation and independent test

We investigated the performance of the GraphSol model on the *eSOL* dataset. As shown in Table 2, We obtained ${\text{R}}^{2}$ values of 0.476 $\pm$ 0.014 and 0.483 for the fivefold cross-validation (CV) and independent test, respectively. When separating the dataset into two discrete states (soluble or not soluble) by a cutoff of 0.5, the Area Under Curve (AUC) values are 0.855 and 0.866 for the fivefold CV and independent test, respectively (Additional file 1: Figure S1). The similar results by the CV and independent test indicated the robustness of the *GraphSol* model.

**Table 2:** The ${\text{R}}^{2}$ between the actual solubility scores and those predicted by *GraphSol* based on individual feature groups, removing each feature group from the final *GraphSol* model, and recursively adding feature groups according to their importance, respectively

Feature groups^a^	CV^d^	Ind. test	Feature groups^b^	CV^d^	Ind. test	Features groups^c^	CV^d^	Ind. test
–	–	–	GraphSol	*0.476 *$\pm$* 0.014*	*0.483*	–	–	–
BLOSUM	0.329 $\pm$ 0.016	0.317	− BLOSUM	0.460 $\pm$ 0.011	0.465	BLOSUM	0.329 $\pm$ 0.016	0.317
AAPHY7	0.293 $\pm$ 0.014	0.289	− AAPHY7	0.465 $\pm$ 0.012	0.479	+ SPIDER3	0.413 $\pm$ 0.012	0.409
PSSM	0.333 $\pm$ 0.012	0.332	− PSSM	0.457 $\pm$ 0.017	0.467	+ PSSM	0.456 $\pm$ 0.011	0.453
HMM	0.337 $\pm$ 0.015	0.341	− HMM	0.455 $\pm$ 0.016	0.458	+ HMM	0.465 $\pm$ 0.012	0.479
SPIDER3	0.231 $\pm$ 0.019	0.227	− SPIDER3	0.428 $\pm$ 0.018	0.449	+ AAPHY7	*0.476 *$\pm$* 0.014*	*0.483*

Italic values indicate the performance of using all feature groups in our model

^a^ Performances based on individual feature groups

^b^ by removing each feature group from all feature

^c^ by adding feature groups recursively

^d^ Performances by the fivefold cross-validation

In order to indicate the importance of feature groups, we assessed the performances by 3 ways in the ablation study. As shown in Table 2, when the individual feature group was used as the node features, the HMM feature yielded the highest ${\text{R}}^{2}$ with a value of 0.341 in the independent test. The other evolution-based feature (PSSM) performed similarly but slightly worse than HMM. Not surprisingly, the BLOSUM feature didn’t perform well with ${\text{R}}^{2}$ of 0.317, but better than the AAPHY7. The predicted structural feature group (SPIDER3) made the worst performance with ${\text{R}}^{2}$ of 0.243. When removing an individual group, on the contrary, the removal of SPIDER3 led to the greatest drop from 0.483 to 0.449. This is likely because SPIDER3 uniquely provided structural information, while other features have supplementary alternatives. Though PSSM and HMM similarly represent evolution information, their removals still caused decreases in performances and generally, HMM is shown more important. The removal of AAPHY7 caused the smallest drop, which is understandable because this feature is a seven-dimensional matrix that is smaller than other feature groups. When we evaluated the model by adding the feature groups recursively, the model showed incremental performances with the addition of each feature group. An interesting fact was that the performance sharply increased from 0.317 to 0.409 after adding SPIDER3 features, which agreed with the relationship between solubility and structural features such as the secondary structure and solvent accessible area.

### Evaluating the impact of predicted protein contact map

**Fig. 2:**
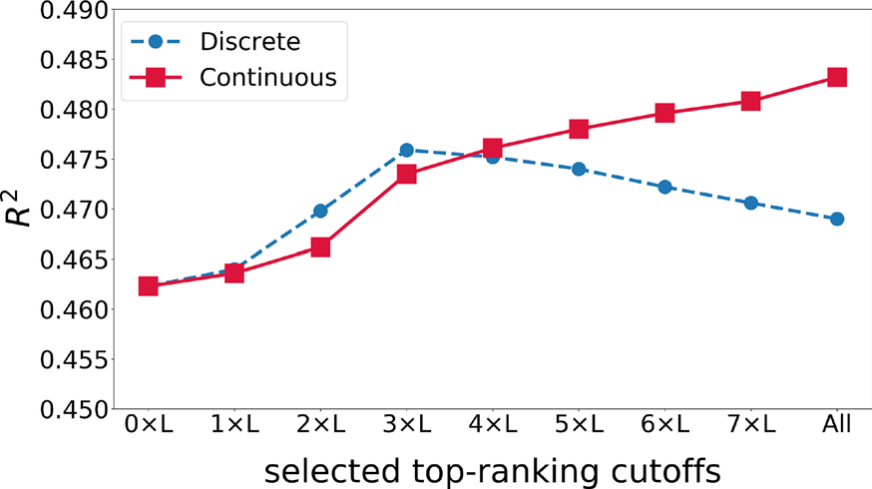
The ${\text{R}}^{2}$ of the GraphSol model changed by selecting the different number of contacts (edges) according to predicted contact maps

The previous results were based on a fully connected graph with edges weighted according to predicted contact probabilities. As there are a limited number of actual contacts between residue pairs, we tested assigning edges between top ${\upalpha }\times \text{L}$ residue pairs with the highest predicted contacting probabilities. As shown in Fig. 2, when not using the predicted contact map (${\upalpha }=0$), i.e. no edges were assigned except between 2-hops neighbored residues, the model achieved ${\text{R}}^{2}$ of 0.462. With the increase of ${\upalpha }$, the ${\text{R}}^{2}$ has a steady increase followed by a sharp increase from $2\times \text{L}$ to $3\times \text{L}$ with the ${\text{R}}^{2}$ of 0.474. Then a slight but continuous growth was observed with an increase of ${\upalpha }$. The highest ${\text{R}}^{2}$ of 0.483 was obtained when all residues were connected with the respective predicted probability.

By comparison, we tested the connectivity by discretely assigning all connected edges as 1 with other pairs not connected and labeled to 0. As expected, the ${\text{R}}^{2}$ increased with ${\upalpha }$ from 0 to 3, indicating that the pairs are helpful for the prediction. Afterward, the ${\text{R}}^{2}$ started to decrease likely due to the increase of inaccurately predicted contact pairs information. Interestingly, we found there are close ${\text{R}}^{2}$ for the discrete group and continuous group at ${\upalpha }$ nearly to 4, which indicates that they may promote equivalent information for the final prediction.

### Comparisons with other methods

**Fig. 3:**
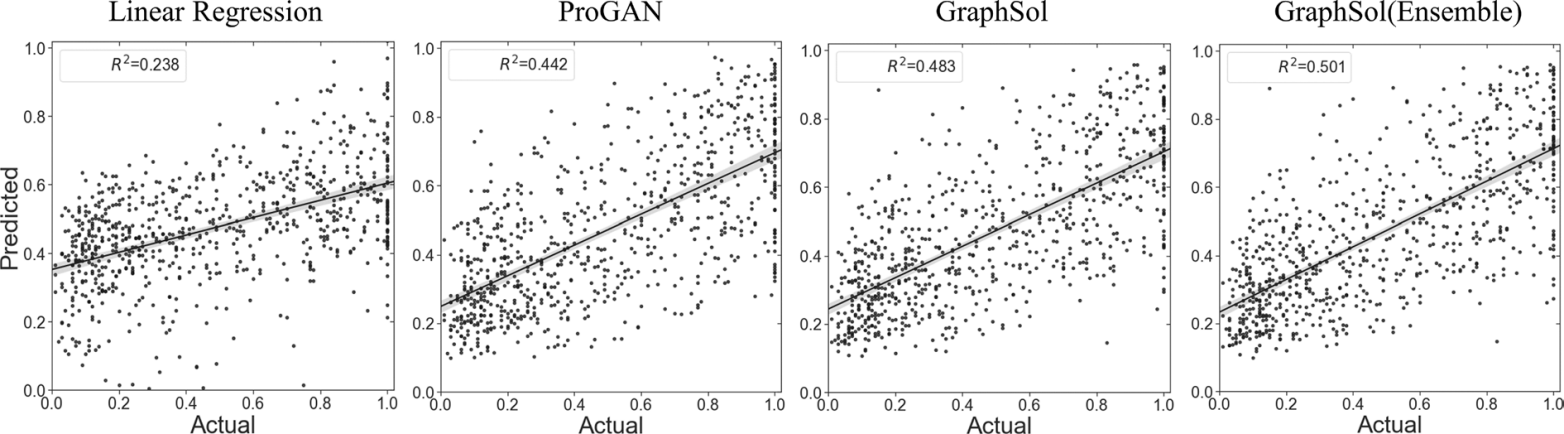
Comparison between actual solubility scores and those predicted by four sequence-based methods on the independent test. The line is the linear least-square fitting to the actual values with the shadow as the 95% confidence intervals for the regressions

Our *GraphSol* model was compared with state-of-the-art methods. To avoid the impacts caused by data, all machine learning or deep learning-based models were retrained and tested on the same train and test dataset, respectively. As shown in Table 2, *GraphSol* consistently obtained the best results by all measurements as a single method with ${\text{R}}^{2}=0.483$. Even if we didn’t use predicted contact maps i.e. no edges were assigned except between 2-hops neighbored residues, the *GraphSol (no-contact)* ranked the 2nd that yielded slightly higher ${\text{R}}^{2}$ than our self-implemented LSTM model (0.462 vs 0.458). This similar result was expected since *GraphSol (no-contact)* utilized the neighbor residue’s information explicitly and the LSTM model obtained the same information implicitly. And SPIDER3 played a key role in providing accurate structural information (0.458 vs 0.449), we inferred this improvement may come from the message in the atom-level rather than the contact in the edge-level. For other methods, TAPE [20] and SeqVec [21], two transfer-learning methods, achieved the highest ${\text{R}}^{2}=0.461$ and the second-highest ${\text{R}}^{2}=0.458$, which lower than *GraphSol* (${\text{R}}^{2}=0.483$) but higher than other non-transfer-learning methods. *ProGAN* [19], a GAN network-based method, achieved ${\text{R}}^{2}=0.442$, which is 4% lower than *TAPE* and *GraphSol(no-contact)*. *DeepSol*, a CNN-based network, achieved ${\text{R}}^{2}$ of 0.434, which is close to the *ProGAN*. Other Machine learning techniques didn’t perform well with ${\text{R}}^{2}$ ranging from 0.214 to 0.411. Figure 3 shows the actual solubility as a function of predicted values by four methods. We found that the deep learning-based methods fitted more accuracy in the region [0.2,0.4], especially the *ProGAN* and *GraphSol* model, and our model performed better in the region of 0.2.

**Fig. 4:**
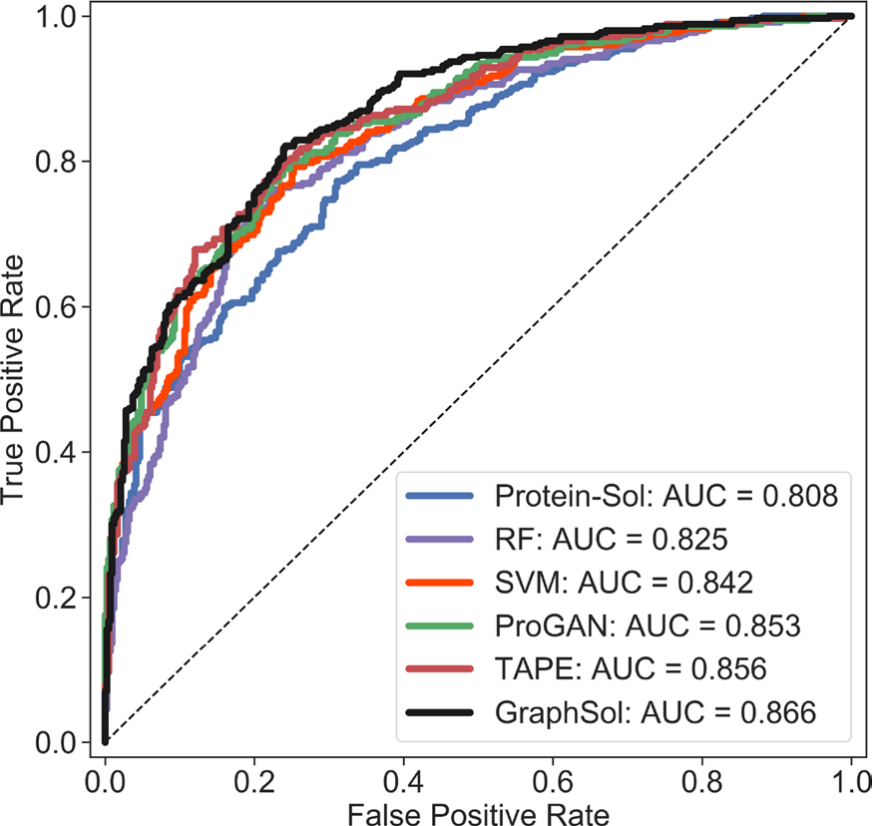
Comparisons of the areas under the receiver operating curve (AUC) on the *eSOL* test set

As most of the other methods were designed for predicting discrete states, we also turn the problem into the 2-state classification task. When using a threshold of 0.5 to define soluble proteins or not, the *GraphSol* model achieved the best performances with an AUC of 0.866, F-measure of 0.732, and accuracy of 0.779, which are at least 2% better than the best of other methods. Figure 4 compared the ROC curves for six methods, and we can find that the curve of *GraphSol* mostly locates on the top. We also tested how accuracy varies as a function of the threshold which defined a soluble class and the trends were similar (Additional file 1: Figures S2, S3).

We noticed that *GraphSol* showed fluctuations in the fivefold cross-validation, and thus we built an ensemble model by averaging the outputs of 5 trained models during CV on the test set. The *GraphSol (Ensemble)* model was found to further improve the performance by a margin of 3% to 0.501 in ${\text{R}}^{2}$. Other indicators also got varied increases (Table 3).

**Table 3:** Comparisons of different methods on the *eSOL* test dataset

Models	RMSE	${\text{R}}^{2}$	Accuracy	Precision	Recall	F1	AUC
K-nearest neighbor	0.284	0.214	0.691	0.737	0.486	0.586	0.776
Linear regression	0.280	0.240	0.707	0.685	0.642	0.663	0.777
Random forest	0.255	0.370	0.760	0.750	0.690	0.729	0.825
Protein-Sol	0.253	0.376	0.714	0.689	0.688	0.693	0.808
XGboost	0.252	0.385	0.756	0.748	0.690	0.718	0.829
Support vector machine	0.246	0.411	0.761	0.763	0.684	0.721	0.842
DeepSol	0.241	0.434	0.763	0.771	0.738	0.695	0.845
ProGAN	0.237	0.442	0.763	0.770	0.676	0.720	0.853
SeqVec	0.236	0.458	0.767	0.754	0.715	0.734	0.858
TAPE	0.235	0.461	0.764	0.774	0.710	0.730	0.856
LSTM (All node features)	0.236	0.458	0.765	0.748	0.677	0.730	0.855
GraphSol (No contact)	0.235	0.462	0.763	0.710	0.676	0.729	0.853
GraphSol	*0.231*	*0.483*	*0.779*	*0.775*	*0.693*	*0.732*	*0.866*
GraphSol (Ensemble)	***0.227***	***0.501***	***0.782***	***0.790***	***0.702***	***0.743***	***0.873***

Italic values indicate the performance of our purposed model

Bold italic values indicate the performance of our ensemble model by using all folds of models to make a final prediction

Moreover, we made comparisons of all methods on the other external *S. cerevisiae* test set. Here, we employed the *eSOL* training dataset to train the model, and have excluded sequences with identity > 25% to the *S. cerevisiae* test dataset. As shown in Table 4, *GraphSol* model yielded ${\text{R}}^{2}$ of 0.358 which is much higher than other sequence-based methods. In comparison, the *DeepSol* and *ProGAN* achieved an ${\text{R}}^{2}$ below 0.1. And our ensemble method could further improve *GraphSol* by 3.9%, consistent with the previous results on the *eSOL* dataset. It is noted that all methods achieved lower ${\text{R}}^{2}$ on this dataset. This is likely because this dataset is more challenging with low homology, as the *Solart* method was reported to yield ${\text{R}}^{2}$ of 0.422 even with the use of experimental structures. This method wasn’t directly compared because most proteins didn’t contain experimental structures as also indicated in the *eSOL* dataset. We also consider the quality of the predicted contact map with the best discrete state $3\times \text{L}$ and ${\text{C}}_{{\upalpha }}$ distance less than 7.5 Å. As a result, the average values of recall and precision are 0.699 and 0.439, respectively. The poor quality of the predicted contact map may lead to the lower ${\text{R}}^{2}$.

**Table 4:** Comparisons of different methods on the S. cerevisiae test set

Solubility predictors	${\text{R}}^{2}$
GraphSol (ensemble)	*0.372*
GraphSol	*0.358*
ccSol^a^	0.302
Protein-Sol^a^	0.281
CamSol^a^	0.160
DeepSol^a^	0.090
ProGAN^b^	0.084

Italic values indicate the best performance of our single model and ensemble model, respectively

^a^Results produced by [7]

^b^Results produced by us

### Case study

**Fig. 5:**
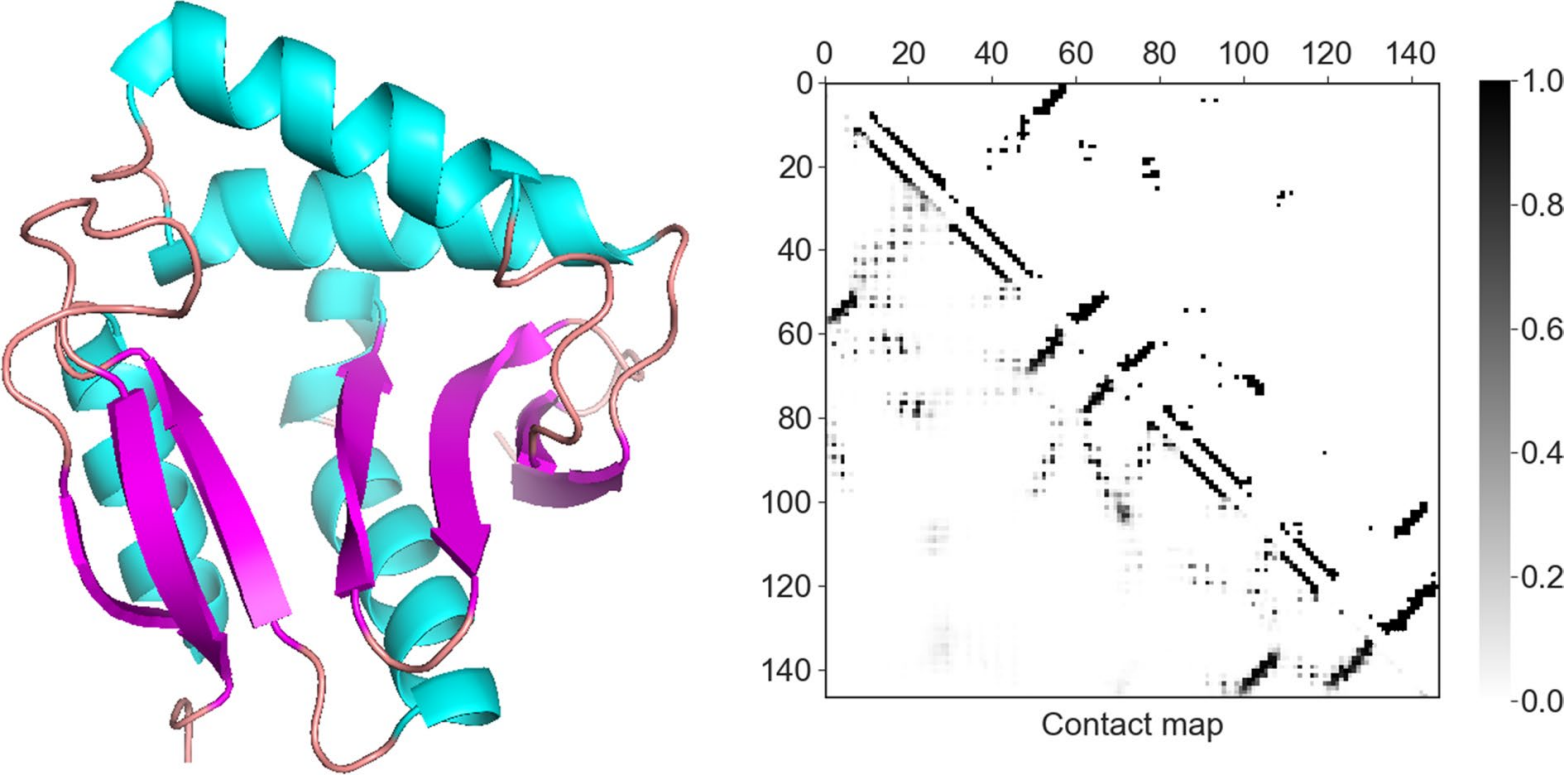
the 3D structure and the predicted (the left down triangular matrix) and actual (the right top one) contact map of the selected case (the Peptidyl-lysine N-acetyltransferase yjaB gene, PDB ID: 2kcw)

To further illustrate our method, we took the Peptidyl-lysine N-acetyltransferase *yjaB* gene (PDB id: 2kcw) that consisted of 147 residues as an example, which showed the lowest RMSE during the independent test. We calculated the ${\text{C}}_{{\upalpha }}$ distance between all residue pairs as the actual contact map. As shown in Fig. 5, there are 360 residue pairs with ${\text{C}}_{{\upalpha }}$ distance less than 7.5 Å on the actual contact map of the protein. The prediction corresponded to a precision of 0.745 to cover 75.3% of actual contacts (Additional file 1: Table S4). This high-quality prediction of the residue pairs enabled the accurate construction of the protein attitude graph and the solubility prediction. Besides, when compared to the actual solubility of 0.87, the *GraphSol* model made an accurate prediction of 0.864 and 0.857 by using the continuous and $3\times \text{L}$ discrete predicted contact map, respectively. This similar tendency between Fig. 2 and Additional file 1: Figure S4 also indicated the effectiveness of our method.

## Conclusions

In this study, we introduce the *GraphSol* model, a novel sequence-based solubility predictor. Compared to other methods, we utilized predicted protein contact maps that played a key role in bridging protein topology attribute and attentive graph neural network. We found that the predicted contact probabilities between residues are better to represent the pairwise relations than discrete states. In the future, such a method is potentially useful to protein attribute predictions including protein function, protein–protein interaction, protein folding, and drug design.

## Supplementary Information

Additional file 1: **Table S1.** The abbreviations list in the paper. **Table S2.** Important hyperparameters were used in the GraphSol model. **Table S3.** The performance of fivefold CV and independent test in the Grid Search of each parameter. **Table S4. **The confused matrix between the predicted contact map and actual contact map of the protein sequence in the case study. **Figure S1.** Comparison of the area under the receiver operating curve (AUC) of GraphSol models on the fivefold CV and independent test. **Figure S2.** Comparison of the precision-recall curve of GraphSol models with the other 5 methods on the independent test. **Figure S3.** Comparison of the accuracy by setting different cutoff for the soluble class with the other 6 methods on the independent test. **Figure S4.** The solubility prediction of the protein sequence that was produced by gene *yjaB*, with the GraphSol model changed by selecting different thresholds according to predicted protein contact maps.

## Data Availability

The source code of *GraphSol* is available at https://github.com/jcchan23/GraphSol.
