# Supplementary material for: Structure-aware protein solubility prediction from sequence through graph convolutional network and predicted contact map
Source: J Cheminform. 2021 Feb 8;13:7. doi: 10.1186/s13321-021-00488-1 (PMC7869490; doi:10.1186/s13321-021-00488-1)
Supplement: Supplementary file 1 — Additional file 1: Table S1. The abbreviations list in the paper. Table S2. Important hyperparameters were used in the GraphSol model. Table S3. The performance of fivefold CV and independent test in the Grid Search of each parameter. Table S4. The confused matrix between the predicted contact map and actual contact map of the protein sequence in the case study. Figure S1. Comparison of the area under the receiver operating curve (AUC) of GraphSol models on the fivefold CV and independent test. Figure S2. Comparison of the precision-recall curve of GraphSol models with the other 5 methods on the independent test. Figure S3. Comparison of the accuracy by setting different cutoff for the soluble class with the other 6 methods on the independent test. Figure S4. The solubility prediction of the protein sequence that was produced by gene yjaB, with the GraphSol model changed by selecting different thresholds according to predicted protein contact maps. [file 13321_2021_488_MOESM1_ESM.docx]

**Additional Information**

**Structure-aware Protein Solubility Prediction from Sequence through**

**Graph Convolutional Network and Predicted Contact map**

Jianwen Chen^1,#^, Shuangjia Zheng^1,#^, Huiying Zhao^2^, Yuedong Yang^1,3,*^

^1^School of Data and Computer Science, Sun Yat-sen University, Guangzhou 510000, China

^2^Sun Yat-sen Memorial Hospital, Sun Yat-sen University, Guangzhou 510000, China

^3^Key Laboratory of Machine Intelligence and Advanced Computing of MOE, Sun Yat-sen University, Guang-zhou 510000, China

^*^To whom correspondence should be addressed.

^#^These two authors contributed equally.

**Contact:** yangyd25@mail.sysu.edu.cn

**Table S1.** The abbreviations list in the paper

| Name | Abbreviations |
| --- | --- |
| Graph neural network | GNN |
| Graph convolutional network | GCN |
| Convolutional neural network | CNN |
| Generative adversarial network | GAN |
| Long short-term memory | LSTM |
| Critical assessment of protein structure prediction | CASP |
| Blocks Substitution Matrix | BLOSUM62 |
| Physicochemical Properties | AAPHY7 |
| Position-specific scoring matrix | PSSM |
| Hidden Markov matrix | HMM |
| Three secondary structure states | SS3 |
| Relative solvent accessible surface area | ASA |
| Half sphere exposures | HSE |

**Table S2.** Important hyperparameters were used in the GraphSol model

| Hyper-Parameters | Description | Ranges | Best value |
| --- | --- | --- | --- |
| Learning Rate | A coefficient to adjust the model weight | 1e-5,1e-4,1e-3 | 1e-4 |
| Weight Decay | A coefficient to avoid overfitting | 2e-4,1e-4,5e-5,1e-5 | 1e-4 |
| GCN Layers | The Graph Convolutional layers that used in the GraphSol model | 1,2,3,4 | 2 |
| GCN Hidden Dimensions | The dimension of the middle GCN layers | 32,64,128,256,512 | 256 |
| GCN Output Dimensions | The dimension of the final GCN layers | 16,32,64,128,256 | 64 |
| Attention Dense Dimensions | The dimension of the final output in the attention pooling layers | 8,16,32,64,128 | 16 |
| Attention Heads | The groups that attention layers used | 1~10 | 4 |

**Table S3.** The performance of 5-fold CV and independent test in the Grid Search of each parameter

(a) Learning Rate

| Hyper Parameters | Value | 5-Fold CV $R^{2}$ | Independent Test $R^{2}$ |
| --- | --- | --- | --- |
| Learning Rate | 1e-3 | 0.469$\pm$0.011 | 0.472 |
|  | **1e-4** | 0.476$\pm$0.014 | 0.483 |
|  | 1e-5 | 0.449$\pm$0.012 | 0.460 |

(b) Weight Decay

| Hyper Parameters | Value | 5-Fold CV $R^{2}$ | Independent Test $R^{2}$ |
| --- | --- | --- | --- |
| Weight Decay | 2e-4 | 0.469$\pm$0.012 | 0.480 |
|  | **1e-4** | 0.476$\pm$0.014 | 0.483 |
|  | 5e-5 | 0.467$\pm$0.011 | 0.478 |
|  | 1e-5 | 0.466$\pm$0.012 | 0.479 |

(c) GCN Layers

| Hyper Parameters | Value | 5-Fold CV $R^{2}$ | Independent Test $R^{2}$ |
| --- | --- | --- | --- |
| GCN Layers | 1 | 0.450$\pm0.012$ | 0.466 |
|  | **2** | 0.476$\pm$0.014 | 0.483 |
|  | 3 | 0.474$\pm$0.011 | 0.481 |
|  | 4 | 0.458$\pm$0.013 | 0.461 |

(d) GCN Hidden Dimensions

| Hyper Parameters | Value | 5-Fold CV $R^{2}$ | Independent Test $R^{2}$ |
| --- | --- | --- | --- |
| GCN Hidden Dimensions | 32 | 0.467$\pm$0.012 | 0.472 |
|  | 64 | 0.466$\pm$0.010 | 0.476 |
|  | 128 | 0.471$\pm$0.013 | 0.480 |
|  | **256** | 0.476$\pm$0.014 | 0.483 |
|  | 512 | 0.468$\pm$0.012 | 0.473 |

(e) GCN Output Dimensions

| Hyper Parameters | Value | 5-Fold CV $R^{2}$ | Independent Test $R^{2}$ |
| --- | --- | --- | --- |
| GCN Output Dimensions | 16 | 0.465$\pm$0.010 | 0.472 |
|  | 32 | 0.467$\pm$0.012 | 0.481 |
|  | **64** | 0.476$\pm$0.014 | 0.483 |
|  | 128 | 0.474$\pm$0.016 | 0.482 |
|  | 256 | 0.469$\pm$0.011 | 0.472 |

(f) Attention Dense Dimensions

| Hyper Parameters | Value | 5-Fold CV $R^{2}$ | Independent Test $R^{2}$ |
| --- | --- | --- | --- |
| Attention Dense Dimensions | 8 | 0.468$\pm$0.012 | 0.476 |
|  | **16** | 0.476$\pm$0.014 | 0.483 |
|  | 32 | 0.466$\pm$0.012 | 0.479 |
|  | 64 | 0.465$\pm$0.010 | 0.472 |
|  | 128 | 0.460$\pm$0.012 | 0.470 |

(g) Attention Heads

| Hyper Parameters | Value | 5-Fold CV $R^{2}$ | Independent Test $R^{2}$ |
| --- | --- | --- | --- |
| Attention Heads | 1 | 0.459$\pm$0.012 | 0.460 |
|  | 2 | 0.463$\pm$0.011 | 0.462 |
|  | 3 | 0.471$\pm$0.010 | 0.473 |
|  | **4** | 0.476$\pm$0.014 | 0.483 |
|  | 5 | 0.470$\pm$0.012 | 0.482 |
|  | 6 | 0.467$\pm$0.012 | 0.477 |
|  | 7 | 0.459$\pm$0.014 | 0.475 |
|  | 8 | 0.461$\pm$0.012 | 0.477 |
|  | 9 | 0.463$\pm$0.010 | 0.476 |
|  | 10 | 0.460$\pm$0.011 | 0.472 |


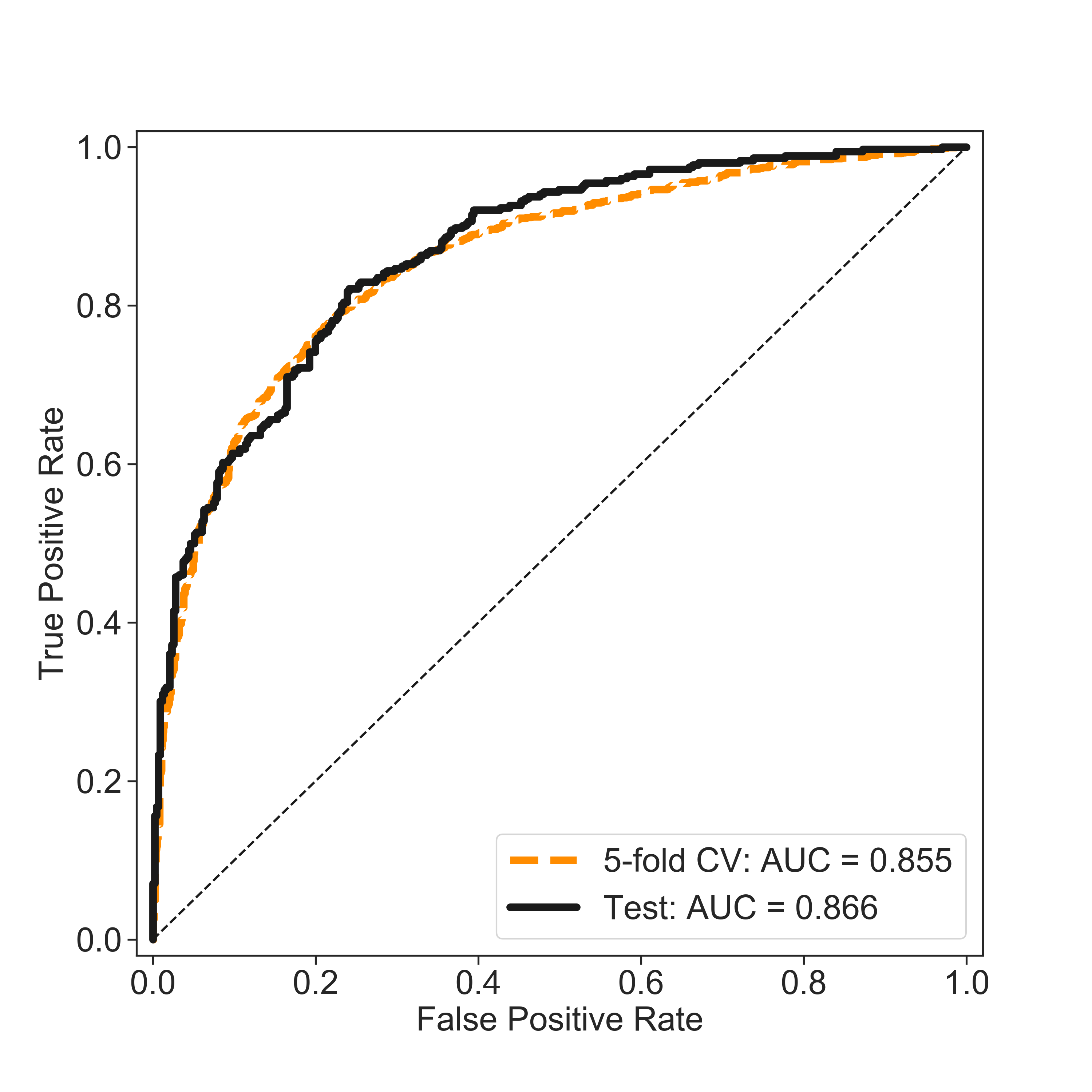


**Figure S1.** Comparison of the area under the receiver operating curve (AUC) of GraphSol models on the 5-fold CV and independent test.


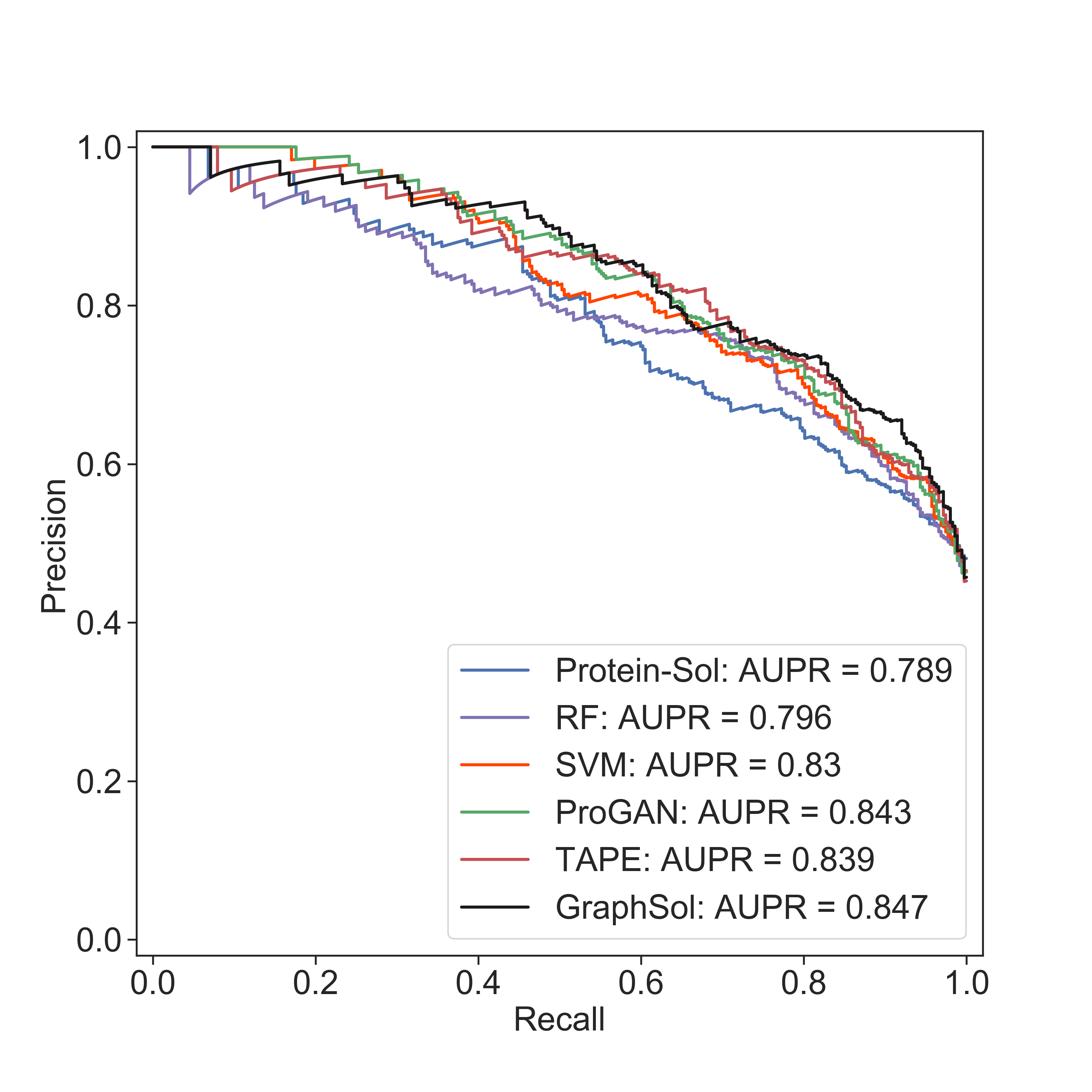


**Figure S2.** Comparison of the precision-recall curve of GraphSol models with the other 6 methods on the independent test.


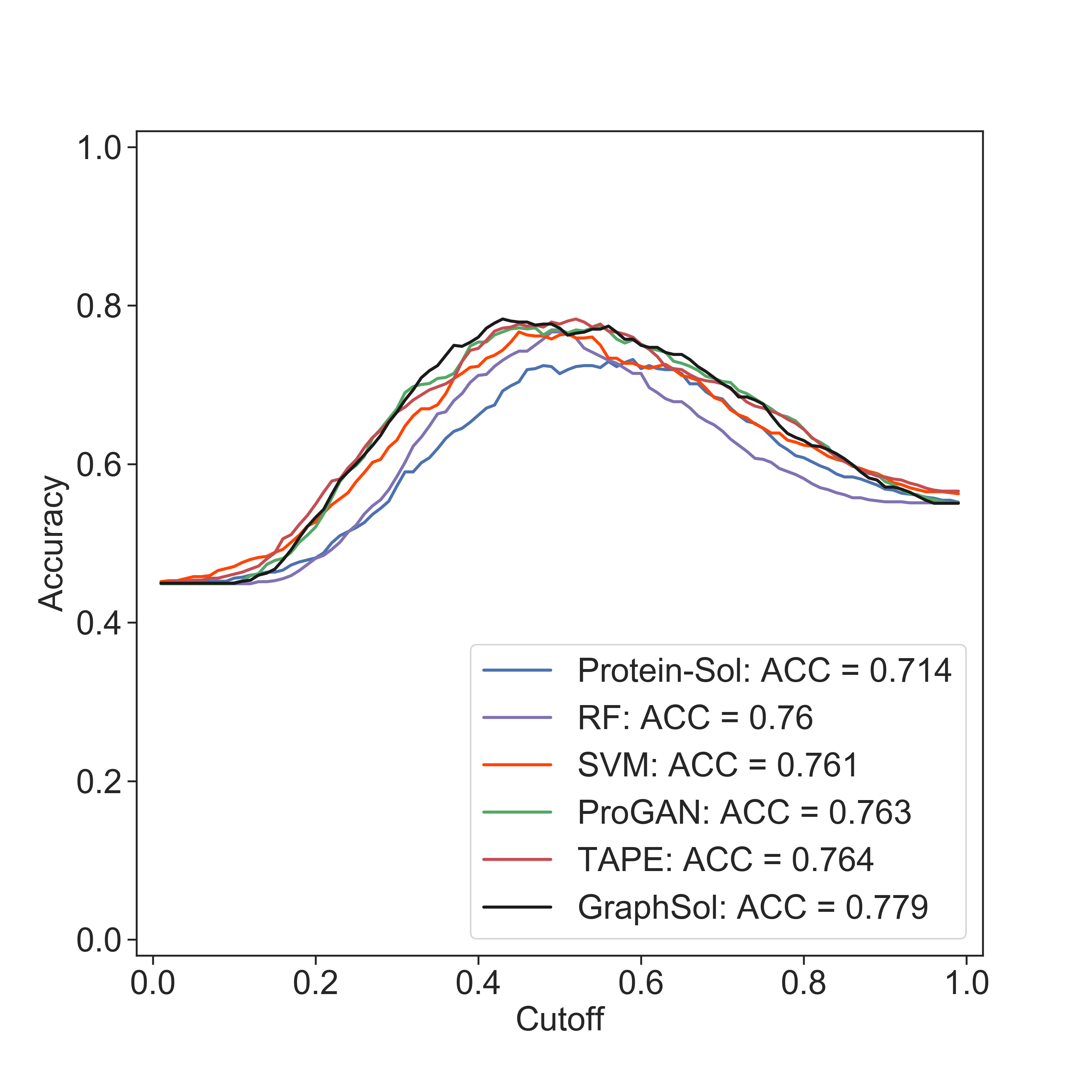


**Figure S3.** Comparison of the accuracy by setting different cutoff for the soluble class with the other 6 methods on the independent test.

**Table S4.** The confused matrix between the predicted contact map and actual contact map of the protein sequence in the case study.

| Predicted\Actual | Contact | Not Contact | Sum |
| --- | --- | --- | --- |
| Contact | $TP=271$ | $FP=93$ | $364$ |
| Not Contact | $FN=89$ | $TN=9987$ | $10076$ |
| Sum | $360$ | $10080$ | $10440$ |

**Note:**

(1) The cutoff of the predicted contact map and actual contact map are 0.5 and 7.5 Å, respectively.

(2) The number of all residue pairs are $147\times147=21609$, we use the residue pairs above the main diagonal because of the symmetry, the number of them is $(21609-147)/2=10731$.

(3) There are some residue pairs that we don’t consider because of locating in the 2-hops neighbors to the main diagonal, the number of them is $2\times\left( 147-2 \right)+1+0=291$.

(4) Therefore, the rest of the residue pairs that we use are $10731-291=10440$, as expected.


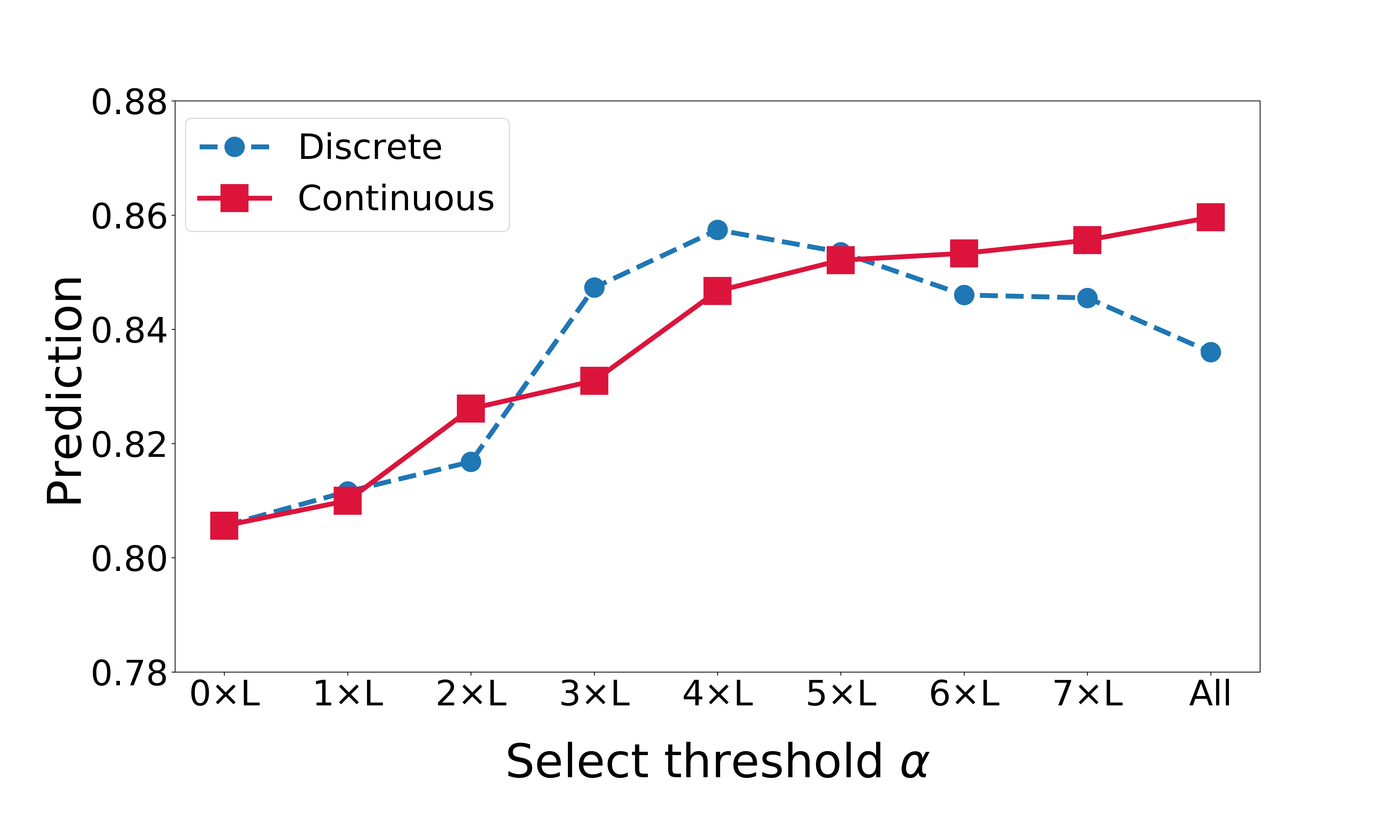


**Figure S4.** The solubility prediction of the protein sequence that produced by gene *yjaB*, with the GraphSol model changed by selecting different thresholds according to predicted protein contact maps.
